# Supplementary material for: Regressive evolution of an effector following a host jump in the Irish potato famine pathogen lineage
Source: PLoS Pathog. 2022 Oct 27;18(10):e1010918. doi: 10.1371/journal.ppat.1010918 (PMC9642902; doi:10.1371/journal.ppat.1010918)
Supplement: S1 Table — PexRD54 and PexRD54-like proteins are listed in the same order (top-bottom) as Fig 1, with the numeric ID corresponding to S2 Fig. For each protein, the clade as determined in the Fig 1 phylogeny (PexRD54, RD54; PexRD54-like, RD54L) is listed. The Phytophthora species, NCBI accession, and length are also recorded for each PexRD54 and PexRD54-like protein. The number of predicted WY domains are noted, based on alignment to the PiPexRD54 sequence [18] and identification of key residues [16]. The aligned amino acid and nucleotide sequences at the PiPexRD54 AIM site are shown. The AIM prediction score from the iLIR software [25] is listed for each amino acid sequence at the PiPexRD54 AIM site, where ‘-‘ denotes no predicted AIM. (PDF) [file ppat.1010918.s001.pdf]

ID	protein clade	species	accession	protein length (AA)	# predicted WY domains	AIM region sequence (AA)	AIM region sequence (nucleotide)	iLIR PSSM score
1	RD54	*P. mirabilis* 3008	NA	381	5	FDWKIV	TTCGACTGGAAAATTGTG	-
2	RD54	*P. mirabilis* P9914	NA	381	5	FDWKIV	TTCGACTGGAAAATTGTG	-
3	RD54	*P. ipomoeae*	NA	381	5	EDWEIV	-	23
4	RD54	*P. infestans* KR_2_A2	KAF4127200.1	349	5	FDWEIV	TTCGACTGGGAAATTGTG	23
5	RD54	*P. infestans* T30-4	XP002903599.1	381	5	FDWEIV	TTCGACTGGGAAATTGTG	23
6	RD54	*P. infestans* KR_1_A1	KAF4033236.1	349	5	FDWEIV	TTCGACTGGGAAATTGTG	23
7	RD54	*P. parasitica* P10297	ETP38918.1	192	3	PDWELV	CCCGATTGGGAATTAGTG	21
8	RD54	*P. parasitica* P1569	ETO69683.1	371	5	PDWELV	CCCGATTGGGAATTAGTG	21
9	RD54	*P. parasitica* INRA-310	XP008894598.1	371	5	PDWELV	CCCGATTGGGAATTAGTG	21
10	RD54	*P. parasitica* race 0	KUG01662.1	371	5	PDWELV	CCCGATTGGGAATTAGTG	21
11	RD54L	*P. mirabilis* 3008	NA	325	5	SEWQFA	TCAGAGTGGCAATTTGCC	-
12	RD54L	*P. infestans* KR_2_A2	KAF4144442.1	484	6	SEWQFA	TCAGAGTGGCAATTTGCC	-
13	RD54L	*P. infestans* T30-4	XP002998586.1	484	6	SEWQFA	TCAGAGTGGCAATTTGCC	-
14	RD54L	*P. infestans* KR_2_A2	KAF4140921.1	406	5	-	-	-
15	RD54L	*P. infestans* KR_1_A1	KAF4034256.1	322	4	-	-	-
16	RD54L	*P. parasitica* P1569	ETI41474.1	482	6	SEWEFA	TCAGAATGGGAGTTTGCA	-
17	RD54L	*P. parasitica* P10297	ETP39360.1	482	6	SEWEFA	TCAGAATGGGAGTTTGCA	-
18	RD54L	*P. parasitica* race 0	KUF68478.1	482	6	SEWEFA	TCAGAATGGGAGTTTGCA	-
19	RD54L	*P. parasitica* INRA-310	XP008894233.1	482	6	SEWEFA	TCAGAATGGGAGTTTGCA	-
20	RD54L	*P. cactorum* 10300	RAW37140.1	472	6	SGWEFA	TCAGGTTGGGAGTTTGCA	-

S1 Table. Characteristics of PexRD54 and PexRD54-like proteins in Fig 1. PexRD54 and PexRD54-like proteins are listed in the same order (top-bottom) as **Fig 1**, with the numeric ID corresponding to **S2 Fig**. For each protein, the clade as determined in the Fig. 1 phylogeny (PexRD54, RD54; PexRD54-like, RD54L) is listed. The *Phytophthora* species, NCBI accession, and length are also recorded for each PexRD54 and PexRD54-like protein. The number of predicted WY domains are noted, based on alignment to the PiPexRD54 sequence (18) and identification of key residues (16). The aligned amino acid and nucleotide sequences at the PiPexRD54 AIM site are shown. The AIM prediction score from the iLIR software (25) is listed for each amino acid sequence at the PiPexRD54 AIM site, where '-' denotes no predicted AIM.
