# Supplementary material for: Regressive evolution of an effector following a host jump in the Irish potato famine pathogen lineage
Source: PLoS Pathog. 2022 Oct 27;18(10):e1010918. doi: 10.1371/journal.ppat.1010918 (PMC9642902; doi:10.1371/journal.ppat.1010918)
Supplement: S2 Table — Table summarizing the thermodynamic and kinetic data for the isothermal titration calorimetry experiments presented in S5 and S6 Figs. (PDF) [file ppat.1010918.s002.pdf]

Peptide	ATG8	Rep.	KD [M]	rM	∆H [cal/mol]	∆G [cal/mol]
PiPexRD54	StATG8-2.2	1	9.39E-08 ± 2.96e-8	1.08 ± 7.70e-3	-3.18E+03 ± 5.11e+1	-9.59E+03 ± 1.87e+2
PiPexRD54	StATG8-2.2	2	1.16E-07 ± 3.19e-8	1.17 ± 4.38e-3	-3.27E+03 ± 3.76e+1	-9.46E+03 ± 1.63e+2
PiPexRD54	StATG8-2.2	3	3.50E-08 ± 1.83e-8	1.45 ± 6.11e-3	-2.85E+03 ± 3.93e+1	-1.02E+04 ± 3.10e+2
PiPexRD54	MjATG8-I	1	2.55E-07 ± 4.13e-8	1.27 ± 8.01e-3	-3.55E+03 ± 2.33e+1	-9.00E+03 ± 9.60e+1
PiPexRD54	MjATG8-I	2	2.41E-07 ± 3.05e-8	1.06 ± 4.50e-3	-3.78E+03 ± 4.10e+1	-9.03E+03 ± 7.51e+1
PiPexRD54	MjATG8-I	3	2.34E-07 ± 5.55e-8	1.44 ± 1.04e-2	-3.28E+03 ± 9.38e+1	-9.05E+03 ± 1.41e+2
PiPexRD54	MjATG8-III	1	1.76E-07 ± 1.19e-8	1.45 ± 1.50e-3	-5.50E+03 ± 1.75e+1	-9.21E+03 ± 4.01e+1
PiPexRD54	MjATG8-III	2	6.63E-08 ± 1.73e-8	1.35 ± 8.04e-3	-5.33E+03 ± 1.06e+1	-9.79E+03 ± 1.55e+2
PiPexRD54	MjATG8-III	3	2.45E-07 ± 2.54e-8	1.51 ± 4.46e-3	-5.99E+03 ± 5.03e+1	-9.02E+03 ± 6.12e+1
PmRD54	StATG8-2.2	1	4.49E-06 ± 2.38e-7	1.23 ± 8.95e-3	-3.23E+03 ± 2.50e+1	-7.30E+03 ± 3.14e+1
PmRD54	StATG8-2.2	2	3.04E-06 ± 5.77e-7	1.35 ± 1.44e-2	-2.34E+03 ± 3.90e+1	-7.53E+03 ± 1.13e+2
PmRD54	StATG8-2.2	3	4.79E-06 ± 5.12e-7	1.27 ± 1.28e-2	-2.98E+03 ± 4.27e+1	-7.26E+03 ± 6.34e+1
PmRD54	MjATG8-I	1	1.56E-05 ± 3.22e-6	1.32 ± 7.2 8e-2	-4.12E+03 ± 3.94e+2	-6.56E+03 ± 1.22e+2
PmRD54	MjATG8-I	2	5.44E-06 ± 5.21e-7	1.00 ± 0.00	-2.74E+03 ± 7.30e+1	-7.18E+03 ± 5.68e+1
PmRD54	MjATG8-III	1	2.04E-05 ± 1.78e-6	1.23 ± 1.89e-2	-3.02E+03 ± 8.34e+1	-6.40E+03 ± 5.16e+1
PmRD54	MjATG8-III	2	1.17E-05 ± 1.95e-6	1.00 ± 0.00	-2.55E+03 ± 1.56e+2	-6.73E+03 ± 9.89e+1
PmRD54	MjATG8-III	3	7.84E-05 ± 2.13e-6	1.00 ± 0.00	-6.93E+03 ± 7.65e+1	-5.60E+03 ± 1.61e+1

S2 Table. Summary of the thermodynamic and kinetic data for the isothermal titration calorimetry experiments. Table summarizing the thermodynamic and kinetic data for the isothermal titration calorimetry experiments presented in **S5 Fig** and **S6 Fig**.
