# Supplementary material for: Regressive evolution of an effector following a host jump in the Irish potato famine pathogen lineage
Source: PLoS Pathog. 2022 Oct 27;18(10):e1010918. doi: 10.1371/journal.ppat.1010918 (PMC9642902; doi:10.1371/journal.ppat.1010918)
Supplement: S3 Table — Table listing all primers used for cloning the constructs used in this study. Amplicon sizes marked with an asterisk (*) are dependent on the vector context. (PDF) [file ppat.1010918.s003.pdf]

Primer name	Sequence (5'-3')	Size (bp)	Usage in this study
**MjATG8-I_F**	*CACCATGGCTAAGAGTTACTTCAAG*		Gateway cloning of MjATG8-I
**MjATG8-I_R**	*CTACACTGTTGCGATCTCCC*	366	Gateway cloning of MjATG8-I
**MjATG8-II_F**	*CACCATGGCCAATAATTCGTTCC*		Gateway cloning of MjATG8-II
**MjATG8-II_R**	*TCAGAAGCCATCGCATTTTGATC*	369	Gateway cloning of MjATG8-II
**MjATG8-III_F**	*CACCATGGTTAAGCCTCAAGTTTTC*		Gateway cloning of MjATG8-III
**MjATG8-III_R**	*TCACCCGCCACCAAAGGTC*	360	Gateway cloning of MjATG8-III
**MjATG8-IV_F**	*CACCATGGCTAAAAGCTCCTTCAAAT*		Gateway cloning of MjATG8-IV
**MjATG8-IV_R**	*TCAGTTAATTCCAAATGCCG*	369	Gateway cloning of MjATG8-IV
**MjATG8-V_F**	*CACCATGGCTAAAAGCTCCTTCAAAT*		Gateway cloning of MjATG8-V
**MjATG8-V_R**	*TCATCCAAACACATTCTCCCC*	351	Gateway cloning of MjATG8-V
**MjATG8-VI_F**	*CACCATGGCTAAAAGCTCTTTCAAGC*		Gateway cloning of MjATG8-VI
**MjATG8-VI_R**	*CTACATGTCTGCACTGCATG*	372	Gateway cloning of MjATG8-VI
**MjATG8-I_pOPINF_F**	*AAGTTCTGTTTCAGGGCCCGGCTAAGAGTTACTTCAAGCA*		In-Fusion cloning of MjATG8-I
**MjATG8-I_pOPINF_R**	*ATGGTCTAGAAAGCTTTACACTGTTGCGATCTCCCCAA*	363	In-Fusion cloning of MjATG8-I
**MjATG8-III_pOPINF_F**	*AAGTTCTGTTTCAGGGCCCGGTTAAGCCTCAAGTTTTCAA*		In-Fusion cloning of MjATG8-III
**MjATG8-III_pOPINF_R**	*TGGTCTAGAAAGCTTTACCCGCCACCAAAGGTCTTTT*	357	In-Fusion cloning of MjATG8-III
**PmPexRD54_F**	*ATGCGTTTCCAGAGCATTATG*		Gateway cloning PmPexRD54
**PmPexRD54_R**	*TTACACAATTTTCCAGTCG*	1146	Gateway cloning PmPexRD54
**sdm-PmPexRD54_PiAIM_F**	*CAAACCGCTGGAGTTCGACTGAAAAATTGTGT*		Site-directed mutagenesis PmPexRD54
**sdm-PmPexRD54_PiAIM_R**	*ACACAATTTTTCAGTCGAACTCCAGCGGTTTG*	*	Site-directed mutagenesis PmPexRD54
**sdm-PiPexRD54_PmAIM_F**	*CCGCTGGATTTCGACTGGGGAATTGTGTAA*		Site-directed mutagenesis PiPexRD54
**sdm-PiPexRD54_PmAIM_R**	*TTACACAATTCCCCAGTCGAAATCCAGCGG*	*	Site-directed mutagenesis PiPexRD54

S3 Table. Primers used in this study. Table listing all primers used for cloning the constructs used in this study. Amplicon sizes marked with an asterisk (*) are dependent on the vector context
