# Supplementary material for: Regressive evolution of an effector following a host jump in the Irish potato famine pathogen lineage
Source: PLoS Pathog. 2022 Oct 27;18(10):e1010918. doi: 10.1371/journal.ppat.1010918 (PMC9642902; doi:10.1371/journal.ppat.1010918)
Supplement: S1 Fig — (a) Phylogeny of Phytophthora clade 1 species was previously reported and the tree depicted here is adapted from Yang et al. 2017 [14]. Species with available genome sequencing data are color-coded corresponding to Fig 1; species without available sequencing data are shown in grey. The Phytophthora subclades (1a, 1b, 1c) are noted. (b) Host specificity of Phytophthora clade 1 species. (PDF) [file ppat.1010918.s004.pdf]

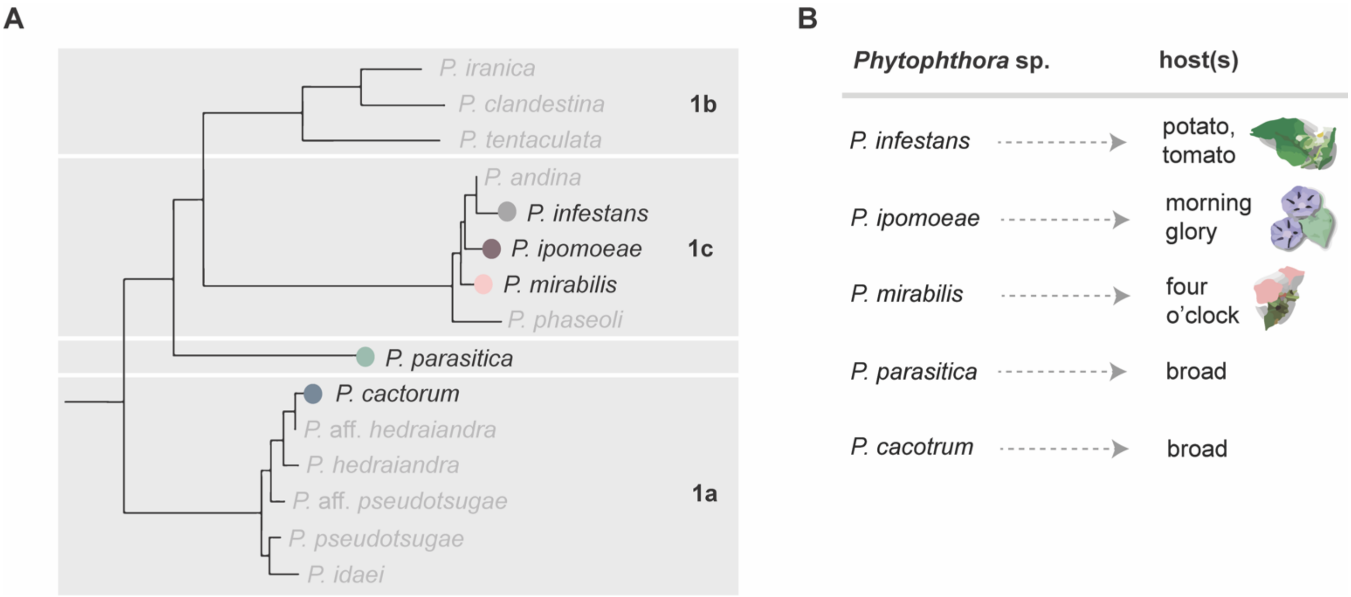

S1 Fig. An overview of the phylogenetic relationships and host range of *Phytophthora* clade 1 species. (a) Phylogeny of *Phytophthora* clade 1 species was previously reported and the tree depicted here is adapted from Yang et al. 2017 (14). Species with available genome sequencing data are color-coded corresponding to **Fig 1**; species without available sequencing data are shown in grey. The *Phytophthora* subclades (1a, 1b, 1c) are noted. **(b)** Host specificity of *Phytophthora* clade 1 species.
