# Supplementary material for: Regressive evolution of an effector following a host jump in the Irish potato famine pathogen lineage
Source: PLoS Pathog. 2022 Oct 27;18(10):e1010918. doi: 10.1371/journal.ppat.1010918 (PMC9642902; doi:10.1371/journal.ppat.1010918)
Supplement: S2 Fig — A 482 amino acid alignment (MUSCLE [25]) of the full-length PexRD54 and PexRD54-like proteins from Fig 1. The proteins are listed in the same order (top-bottom) as Fig 1, with the numeric ID [1–20] corresponding to the key and to Table S1. The predicted WY domain boundaries were mapped based on the PiPexRD54 sequence (WY-1 –WY-5) [18] and identification of key residues based on the WY domain MEME (WY-6) [16]. The RxLR-dEER motif is noted, as is the location of the PexRD54 C-terminal AIM site. (PDF) [file ppat.1010918.s005.pdf]

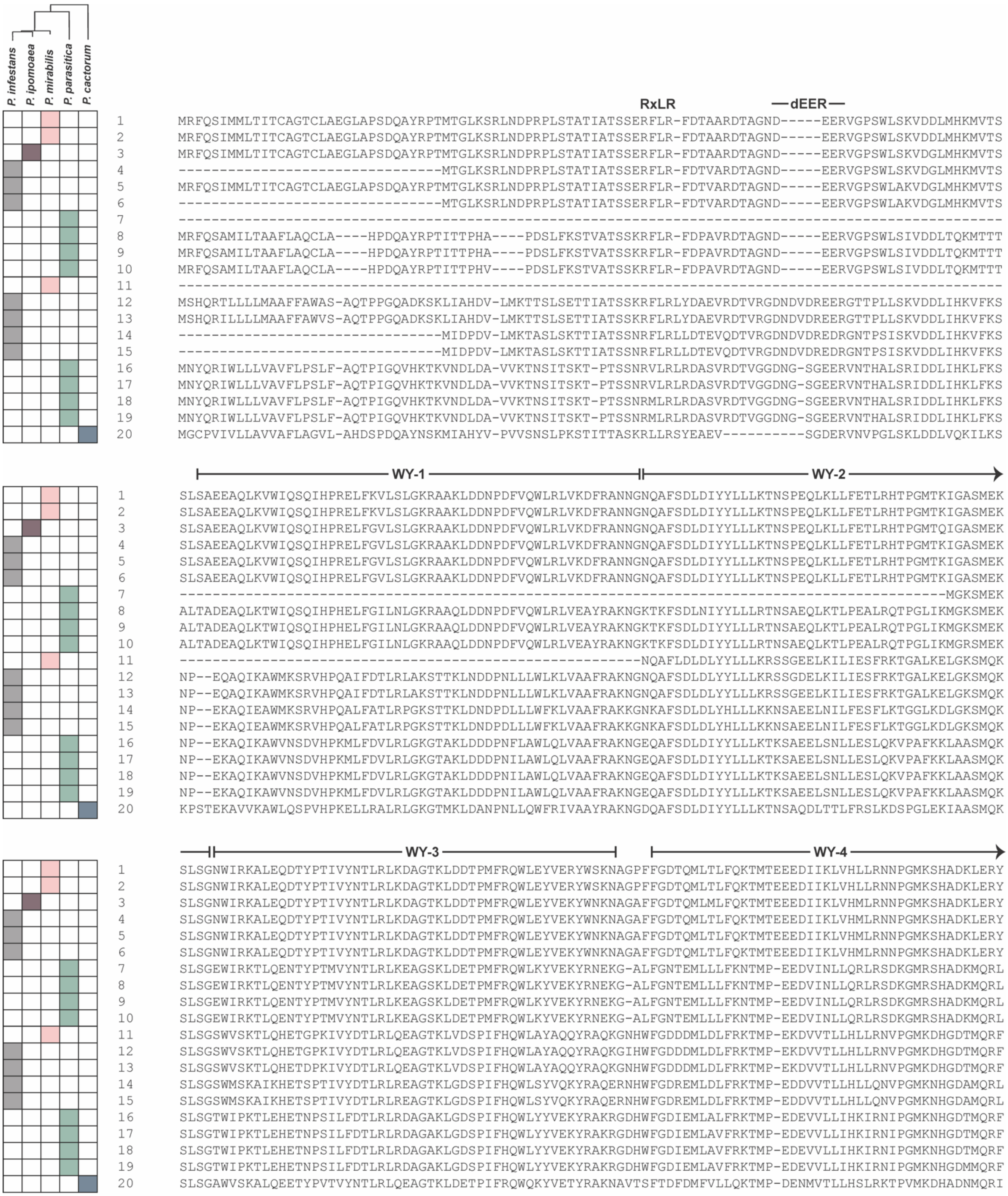
P. infestans
P. ipomoeaea
P. mirabilis
P. parasitica
P. cactorum
RxLR
dEER
WY-1
WY-2
WY-3
WY-4

CONTINUED

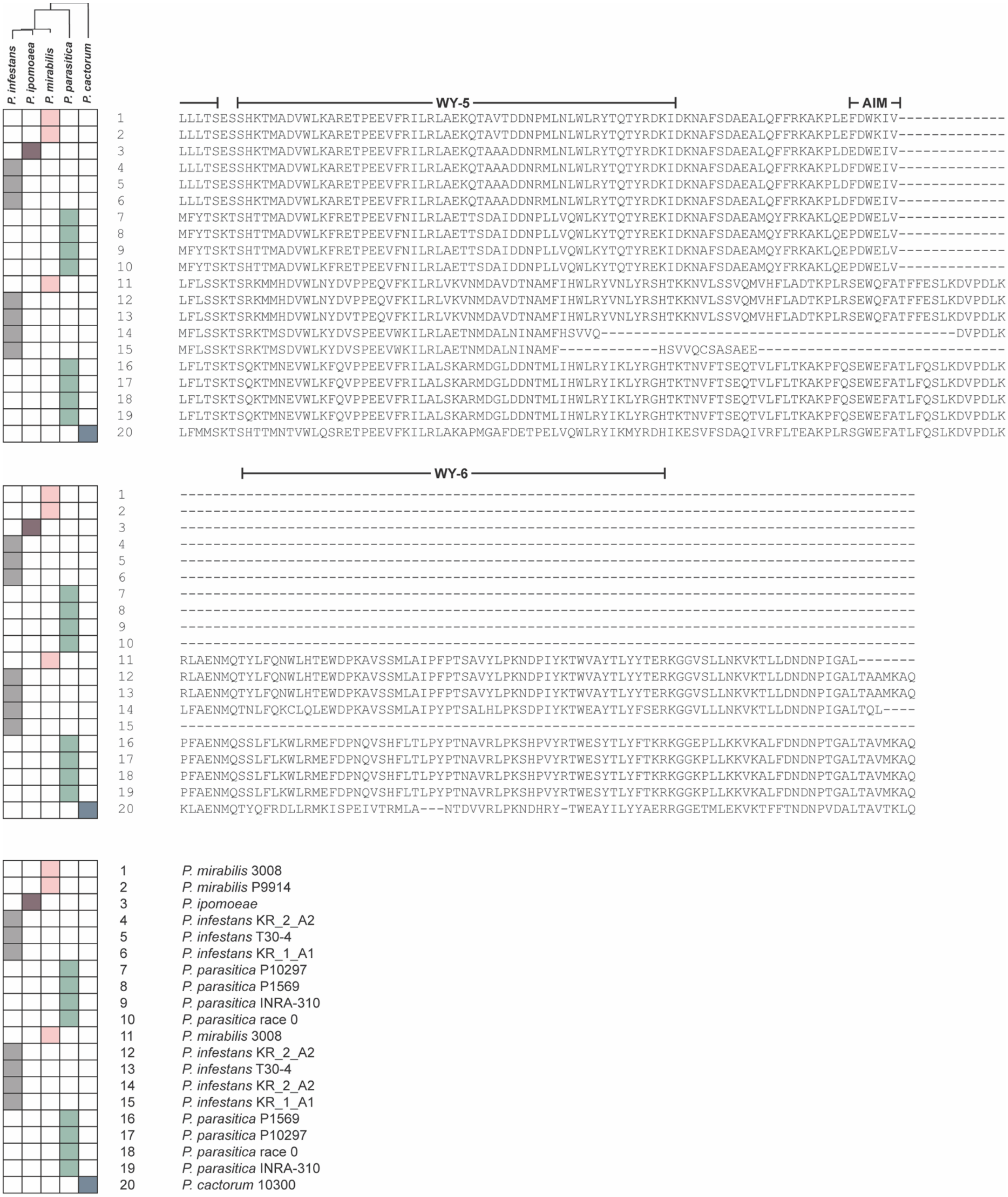
P. infestans
P. ipomoeae
P. mirabilis
P. parasitica
P. cactorum
WY-5
AIM
1 LLLTSESSHKTMADVWLKARETPEEVFRILRLAEKQTAVTDDNPMLNLWLRYTQTYRDKIDKNAFSDAEALQFFRKAKPLEFDWKIV-------------
2 LLLTSESSHKTMADVWLKARETPEEVFRILRLAEKQTAVTDDNPMLNLWLRYTQTYRDKIDKNAFSDAEALQFFRKAKPLEFDWKIV-------------
3 LLLTSESSHKTMADVWLKARETPEEVFRILRLAEKQTAAADDNRMLNLWLRYTQTYRDKIDKNAFSDAEALQFFRKAKPLDEDWEIV-------------
4 LLLTSESSHKTMADVWLKARETPEEVFRILRLAEKQTAAADDNRMLNLWLRYTQTYRDKIDKNAFSDAEALQFFRKAKPLDFDWEIV-------------
5 LLLTSESSHKTMADVWLKARETPEEVFRILRLAEKQTAAADDNRMLNLWLRYTQTYRDKIDKNAFSDAEALQFFRKAKPLDFDWEIV-------------
6 LLLTSESSHKTMADVWLKARETPEEVFRILRLAEKQTAAADDNRMLNLWLRYTQTYRDKIDKNAFSDAEALQFFRKAKPLDFDWEIV-------------
7 MFYTSKTSHTTMADVWLKFRETPEEVFNILRLAETTSDAIDDNPLLVQWLKYTQTYREKIDKNAFSDAEAMQYFRKAKLQEPDWELV-------------
8 MFYTSKTSHTTMADVWLKFRETPEEVFNILRLAETTSDAIDDNPLLVQWLKYTQTYREKIDKNAFSDAEAMQYFRKAKLQEPDWELV-------------
9 MFYTSKTSHTTMADVWLKFRETPEEVFNILRLAETTSDAIDDNPLLVQWLKYTQTYREKIDKNAFSDAEAMQYFRKAKLQEPDWELV-------------
10 MFYTSKTSHTTMADVWLKFRETPEEVFNILRLAETTSDAIDDNPLLVQWLKYTQTYREKIDKNAFSDAEAMQYFRKAKLQEPDWELV-------------
11 LFLSSKTSRKMMHDVWLNYDVPPEQVFKILRLVKVNMDAVDTNAMFIHWLRYVNLYRSHTKKNVLSSVQMVHFLADTKPLRSEWQFATFFESLKDVPDLK
12 LFLSSKTSRKMMHDVWLNYDVPPEQVFKILRLVKVNMDAVDTNAMFIHWLRYVNLYRSHTKKNVLSSVQMVHFLADTKPLRSEWQFATFFESLKDVPDLK
13 LFLSSKTSRKMMHDVWLNYDVTPEQVFKILRLVKVNMDAVDTNAMFIHWLRYVNLYRSHTKKNVLSSVQMVHFLADTKPLRSEWQFATFFESLKDVPDLK
14 MFLSSKTSRKTMSDVWLKYDVSPEEVWKILRLAETNMDALNINAMFHSVVQ--DVPDLK
15 MFLSSKTSRKTMSDVWLKYDVSPEEVWKILRLAETNMDALNINAMF-----------HSVVQCSASAEE------------------------------
16 LFLTSKTSQKTMNEVWLKFQVPPEEVFRILALSKARMDGLDDNTMLIHWLRYIKLYRGHTKTNVFTSEQTVLFLTKAKPFQSEWEFATLFQSLKDVPDLK
17 LFLTSKTSQKTMNEVWLKFQVPPEEVFRILALSKARMDGLDDNTMLIHWLRYIKLYRGHTKTNVFTSEQTVLFLTKAKPFQSEWEFATLFQSLKDVPDLK
18 LFLTSKTSQKTMNEVWLKFQVPPEEVFRILALSKARMDGLDDNTMLIHWLRYIKLYRGHTKTNVFTSEQTVLFLTKAKPFQSEWEFATLFQSLKDVPDLK
19 LFLTSKTSQKTMNEVWLKFQVPPEEVFRILALSKARMDGLDDNTMLIHWLRYIKLYRGHTKTNVFTSEQTVLFLTKAKPFQSEWEFATLFQSLKDVPDLK
20 LFMMSKTSHTTMNTVWLQSRETPEEVFKILRLAKAPMGAFDETPELVQWLRYIKMYRDHIKESVFSDAQIVRFLTEAKPLRSGWEFATLFQSLKDVPDLK
WY-6
1 ---
2 ---
3 ---
4 ---
5 ---
6 ---
7 ---
8 ---
9 ---
10 ---
11 RLAENMQTYLFQNWLHTEWDPKAVSSMLAIPFPTSAVYLPKNDPIYKTWVAYTLYYTERKGGVSLLNKVKTLLDNDNPIGAL-------
12 RLAENMQTYLFQNWLHTEWDPKAVSSMLAIPFPTSAVYLPKNDPIYKTWVAYTLYYTERKGGVSLLNKVKTLLDNDNPIGALTAAMKAQ
13 RLAENMQTYLFQNWLHTEWDPKAVSSMLAIPFPTSAVYLPKNDPIYKTWVAYTLYYTERKGGVSLLNKVKTLLDNDNPIGALTAAMKAQ
14 LFAENMQTNLFQKCLQLEWDPKAVSSMLAIPYPTSALHLPKSDPIYKTWEAYTLYFSERKGGVLLLNKVKTLLDNDNPIGALTQL----
15 ---
16 PFAENMQSSLFLKWLRMEFDPNQVSHFLTLPYPTNAVRLPKSHPVYRTWESYTLYFTKRKGGEPLLKKVKALFDNDNPTGALTAVMKAQ
17 PFAENMQSSLFLKWLRMEFDPNQVSHFLTLPYPTNAVRLPKSHPVYRTWESYTLYFTKRKGGKPLLKKVKALFDNDNPTGALTAVMKAQ
18 PFAENMQSSLFLKWLRMEFDPNQVSHFLTLPYPTNAVRLPKSHPVYRTWESYTLYFTKRKGGKPLLKKVKALFDNDNPTGALTAVMKAQ
19 PFAENMQSSLFLKWLRMEFDPNQVSHFLTLPYPTNAVRLPKSHPVYRTWESYTLYFTKRKGGKPLLKKVKALFDNDNPTGALTAVMKAQ
20 KLAENMQTYQFRDLLRMKISPEIVTRMLA---NTDVVRLPKNDHRY-TWEAYILYYAERRGGETMLEKVKTFFTNDNPVDALTAVTKLQ
1 P. mirabilis 3008
2 P. mirabilis P9914
3 P. ipomoeae
4 P. infestans KR_2_A2
5 P. infestans T30-4
6 P. infestans KR_1_A1
7 P. parasitica P10297
8 P. parasitica P1569
9 P. parasitica INRA-310
10 P. parasitica race 0
11 P. mirabilis 3008
12 P. infestans KR_2_A2
13 P. infestans T30-4
14 P. infestans KR_2_A2
15 P. infestans KR_1_A1
16 P. parasitica P1569
17 P. parasitica P10297
18 P. parasitica race 0
19 P. parasitica INRA-310
20 P. cactorum 10300

S2 Fig. Full-length alignment of PexRD54 and PexRD54-like proteins from Fig 1. A 482 amino acid alignment (MUSCLE (25)) of the full-length PexRD54 and PexRD54-like proteins from **Fig 1**. The proteins are listed in the same order (top-bottom) as **Fig 1**, with the numeric ID (1-20) corresponding to the key and to Table S1. The predicted WY domain boundaries were mapped based on the PiPexRD54 sequence (WY-1 – WY-5) (18) and identification of key residues based on the WY domain MEME (WY-6) (16). The RxLR-dEER motif is noted, as is the location of the PexRD54 C-terminal AIM site.
