# Supplementary material for: Regressive evolution of an effector following a host jump in the Irish potato famine pathogen lineage
Source: PLoS Pathog. 2022 Oct 27;18(10):e1010918. doi: 10.1371/journal.ppat.1010918 (PMC9642902; doi:10.1371/journal.ppat.1010918)
Supplement: S3 Fig — Graph representing the transcript abundance for PmPexRD54 and the control elongation factor 1-alpha (EF1a) in P. mirabilis strain 09316 mycelia and 2–6 days post infection (dpi) of M. jalapa, across three technical replicates. Transcript abundance was measured by RNAseq and is reported in transcripts per million (TPM). (PDF) [file ppat.1010918.s006.pdf]

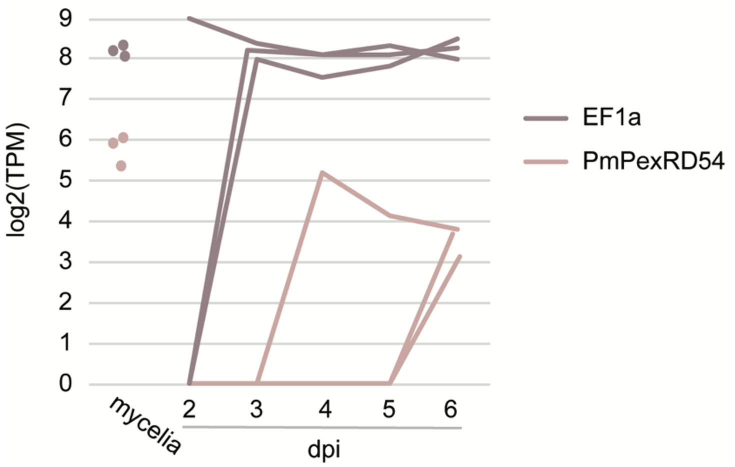

S3 Fig. PmPexRD54 is expressed during *P. mirabilis* infection of *M. jalapa*. Graph representing the transcript abundance for PmPexRD54 and the control elongation factor 1-alpha (EF1a) in *P. mirabilis* strain 09316 mycelia and 2–6 days post infection (dpi) of *M. jalapa*, across three technical replicates. Transcript abundance was measured by RNAseq and is reported in transcripts per million (TPM).
