# Supplementary material for: Regressive evolution of an effector following a host jump in the Irish potato famine pathogen lineage
Source: PLoS Pathog. 2022 Oct 27;18(10):e1010918. doi: 10.1371/journal.ppat.1010918 (PMC9642902; doi:10.1371/journal.ppat.1010918)
Supplement: S4 Fig — Unrooted maximum-likelihood tree of 186 ATG8 isoforms, with clades marked and colored as in Fig 2B. The tree was calculated in MEGA7 [26] from a 445 nucleotide alignment (MUSCLE [25], codon-based). The Solanales and Brassicales ATG8 clades are named following the conventions in Kellner et al. 2017 [29]. The bootstrap values of the major nodes are indicated. The scale bar indicates the evolutionary distance based on nucleotide substitution rate. (PDF) [file ppat.1010918.s007.pdf]

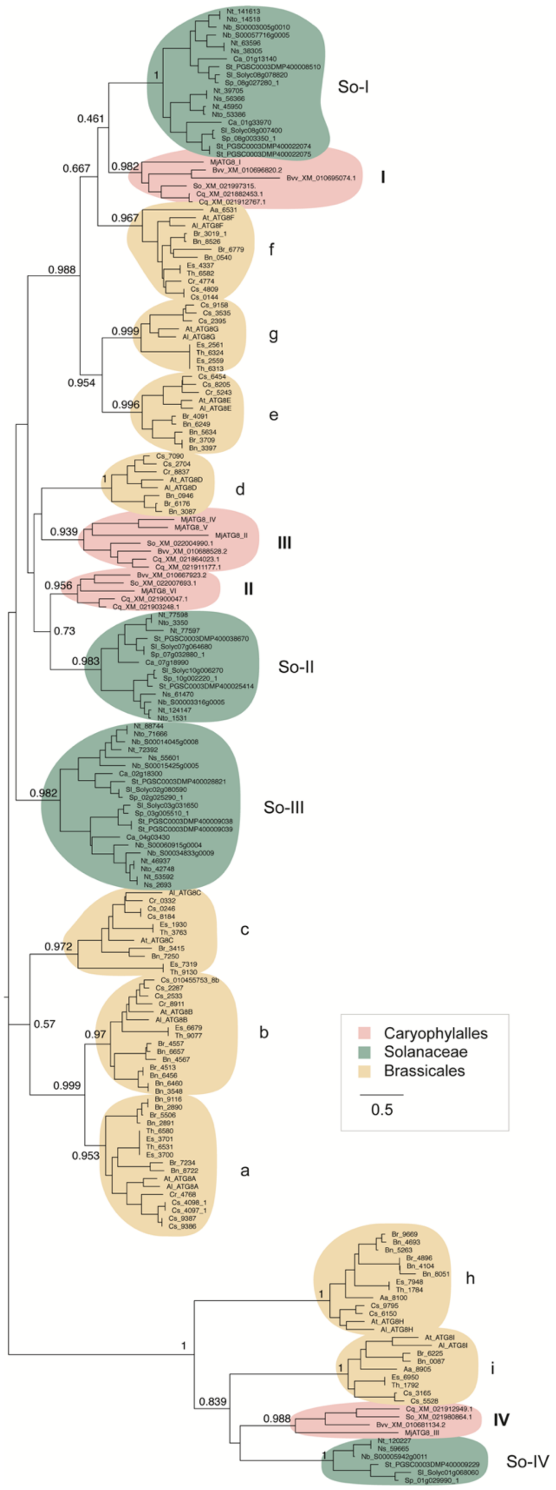

S4 Fig. Phylogenetic relationship between ATG8s from the Caryophyllalles, Solanales, and Brassicales. Unrooted maximum-likelihood tree of 186 ATG8 isoforms, with clades marked and colored as in **Fig 2B**. The tree was calculated in MEGA7 (26) from a 445 nucleotide alignment (MUSCLE (25), codon-based). The Solanales and Brassicales ATG8 clades are named following the conventions in Kellner et al. 2017 (29). The bootstrap values of the major nodes are indicated. The scale bar indicates the evolutionary distance based on nucleotide substitution rate.
