# Supplementary material for: Regressive evolution of an effector following a host jump in the Irish potato famine pathogen lineage
Source: PLoS Pathog. 2022 Oct 27;18(10):e1010918. doi: 10.1371/journal.ppat.1010918 (PMC9642902; doi:10.1371/journal.ppat.1010918)
Supplement: S5 Fig — The binding affinities between PiPexRD54 AIM peptide and StATG8-2.2, MjATG8-I, and MjATG8-III were determined using isothermal titration calorimetry (ITC). The top panels show heat differences upon injection of peptide ligands, and the lower panels show integrated heats of injection (•) and the best fit (pink line) to a single site binding model using AFFINImeter analysis software [32]. (PDF) [file ppat.1010918.s008.pdf]

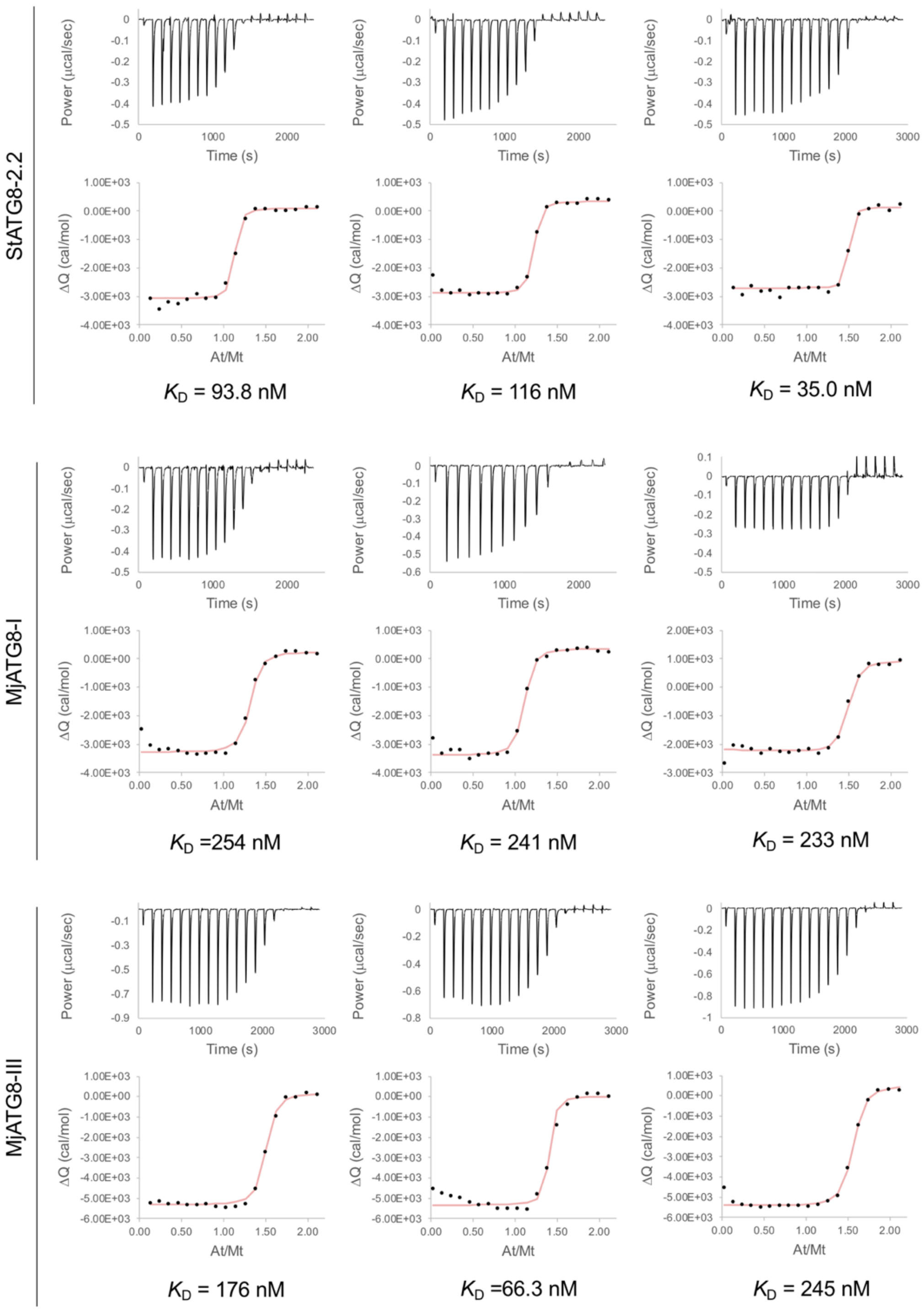

S5 Fig. PiPexRD54 AIM peptide interaction with StATG8-2.2, Mj**ATG8-I and** Mj**ATG8-III in isothermal titration calorimetry.** The binding affinities between PiPexRD54 AIM peptide and StATG8-2.2, MjATG8-I, and MjATG8-III were determined using isothermal titration calorimetry (ITC). The top panels show heat differences upon injection of peptide ligands, and the lower panels show integrated heats of injection (•) and the best fit (pink line) to a single site binding model using AFFINImeter analysis software (32).
