# Supplementary material for: Regressive evolution of an effector following a host jump in the Irish potato famine pathogen lineage
Source: PLoS Pathog. 2022 Oct 27;18(10):e1010918. doi: 10.1371/journal.ppat.1010918 (PMC9642902; doi:10.1371/journal.ppat.1010918)
Supplement: Attachment — Submitted filename: PmRD54_RESPONSE.pdf [file ppat.1010918.s010.pdf]

Norwich Research Park
Norwich NR4 7UH
United Kingdom
+44 (0)1603 450 400
admin@TSL.ac.uk
www.TSL.ac.uk

**Responses to Reviewers**

**Part I - Summary**

Reviewer #1: In the paper "Regressive evolution of an effector following a host jump in the Irish Potato Famine Pathogen Lineage", the authors the explore the evolution of the PexRD54 protein encoding gene within clade 1b and 1c of Phytophthora. The experiments are interesting and suitable, and the conclusions seem largely well supported, and provide an interesting new story on the evolution of these important pathogens. Overall, I enjoyed reading this thought-provoking paper.

RESPONSE: We thank the reviewer for these positive comments.

Reviewer #2: The MS (PPATHOGENS-D-22-00023) is a continuation of research from this group that studies host jump and RXLR effector PexRD54 of Phytophthora infestans. Previously, they presented that a single polymorphism in the particular effectors may undergo adaption to host environments in the cases of Phytophthora infestans or Magnaporthe oryzae and other similar clade pathogens. They have also shown that PiPexRD54 may interact with plant ATG8 protein family via its C-terminal AIM sequence to interfere with the normal operation of host autophagy pathway. In this MS, the group presents PiPexRD54 from P. infestans and PmPexRD54 from P. mirabilis as another example for host specificity, and shows that a single amino acid polymorphism at AIM exhibit different binding activities with ATG8s from their corresponding host plants. Interestingly, PmPexRD54 AIM peptide bind very weakly to the M. jalapa ATG8s. Based on these results, the authors propose a model of regressive evolution of an effector in host jump. The MS is very well prepared with clear results and well-designed experiments. I like this kind of short and significant story very much.

RESPONSE: We also thank this reviewer for the positive comments and overall appreciative evaluation.

1.What is the real function of PmPexRD54 in P. mirabilis infection? Transient expression of PiPexRD54 and PmPexRD54 in Nicotiana benthamiana is desirable.

RESPONSE: We haven't yet identified the function of PmPexRD54 in part because of the limitations of the Mirabilis host plant. As we show in the manuscript, we have already expressed both PiPexRD54 and PmPexRD54 in Nicotiana benthamiana and focus on the differential activities of these orthologous effectors. Therefore, the focus of the paper was to emphasize the concept of regressive evolution. The function of PmPexRD54 in its host plant Mirabilis is beyond this focus.

2.Does PmPexRD54 also interfere with plant autophagy pathway, which is comparable with PiPexRD54 in previous reports or not?

RESPONSE: As we described in the paper, PmPexRD54 doesn't bind to ATG8 proteins and therefore doesn't interfere with host autophagy like PiPexRD54 does.

3.As their previous papers, any amino acid substitution except Pro at position 379 does not alter interaction PexRD54 with ATG8s. It is possible to predict and compare the structure modeling of interaction between PmPexRD54-ATG8s and PiPexRD54-ATG8s?

Norwich Research Park
Norwich NR4 7UH
United Kingdom
+44 (0)1603 450 400
admin@TSL.ac.uk
www.TSL.ac.uk

RESPONSE: Structural modelling of PexRD54-ATG8 complexes failed to yield any solid insights beyond the described differences on the AIM peptide. Our conclusion is that the PmPexRD54 AIM peptide is degenerated.

4.How about other RXLR effectors with AIM in P. mirabilis? Does it have?

RESPONSE: This would be highly speculative. The AIM sequence is fairly short and is frequently predicted in effectors (and other proteins) with a high rate of false positives. In fact, in the original PiPexRD54 paper we observed at least two predicted AIM sequences but only one of them turned out to be functional.

Reviewer #3: Host jump is a major model that pathogens expand or switch their host range. However, the process of pathogen–host specialization is remaining largely unknown. In this manuscript, the authors identified the AIM motif in the effector PexRD54 has degenerated following a host jump in a Phytophthora lineage". This is an interesting story. The authors showed a good example that how pathogen evolves by losing molecular function to adapt new host environment.

RESPONSE: We are pleased that this reviewer is also positive and highlights that our work contributes to understanding the process of pathogen–host specialization.

---

**Part II – Major Issues: Key Experiments Required for Acceptance**

Reviewer #1: I don't think any of the issues I have are major (apart from perhaps the control in the co-IP suggestion that can perhaps be reasonably explained), and should mostly be reasonably quick to address either in the wording/text. I will therefore list them all below in the 'minor issues'.

RESPONSE: We have addressed the reviewer comment as indicated below.

Reviewer #2: Same as above.

RESPONSE: Please see above.

Reviewer #3: (No Response)

RESPONSE: Thank you.

---

**Part III – Minor Issues: Editorial and Data Presentation Modifications**

Reviewer #1: Figure S1 is currently not a very informative phylogenetic tree. The legend does not specify how the tree was constructed, there is no scale bar, or indication of what the branch lengths show, and no details of confidence or support such a bootstraps. They mention its taken from another paper, but all of this information should be briefly mentioned in the legend, and described in further detail in the "Phylogenetic analyses" methods section of this paper. I think these are important details for interpreting every tree, but are particularly important given the very small branch lengths between the species being discussed.

Norwich Research Park
Norwich NR4 7UH
United Kingdom
+44 (0)1603 450 400
admin@TSL.ac.uk
www.TSL.ac.uk

RESPONSE: We disagree with the reviewer's comment. Figure S1 basically sets the stage for the paper given that it describes the evolutionary framework under which we have set up our study. The tree is indeed adapted from previous studies, which have all reported consistent phylogenies. It would be beyond the scope of this paper to redo the Phytophthora phylogeny, and in any case our own analyses are consistent with this tree. I suppose if we provide an original tree we would be criticized for ignoring the previous papers. In any case, we modified the text to make these points more explicit.

"Of these species, P.infestans ... evolved from a series of recent host jumps". I have two questions about this: 1) Is there any dating done to ascertain the timing, or how do you know it is recent (and what is meant by recent)? It would be good to provide further details about what is known regarding this. 2) Given that some species of clade 1b and 1c have a broad host-range, as mentioned in Fig S1 and elsewhere, is it not possible that the ancestor also had a broad-host range, and that some of these current pathogens have specialised, rather than jumped host? Can the authors provide further information to clarify this point? And potentially clarify if necessary throughout (intro, discussion etc).

RESPONSE: 1) We have refrained from dating the Phytophthora tree because the absence of robust timepoints (sampling time series, fossil evidence etc.). We remain sceptical of the robustness of previously reported estimates. However, to respond to the reviewer's point, the sister species are indeed very close, for example the ITS sequences are quasi-identical with very few SNPs at the most. 2) Yes, this is of course possible and we have added a comment to address the point. However, given that all clade 1c species are host-specialized this broad host-range common ancestor most probably predates the emergence of clade 1c as discussed in earlier literature.

"possibly due to sequencing errors" – such as? Why have the PexRF54 sequence for P. paristica been taken from transcriptome data? Is there no genome assembly for it? In addition to causing a truncation, might there be other issues such as splicing, mis-assembly or reduced quality? Might this prove an issue for the downstream analysis and interpretation of this sequence?

RESPONSE: The reviewer makes a good point and we rephrased to clarify this point. Indeed, the gene models couldn't be validated using the available genome data.

"This data falsified our initial hypothesis, and we conclude.". A very minor thought about this, is a concern about writing your a priori hypothesis in this paragraph as a central topic (starting and finishing the paragraph). It might be better to simply state what the results showed or suggested, rather than what you thought was going to happen before the experiment.

RESPONSE: We thank the reviewer for this point and have reworded the text accordingly.

Regarding the co-IP, was there a reason for not including P. ipomoea or P. infestans PexRD54 (that do have AIM predicted domains) and their ATG8 targets? (to make sure the experiment is working as expected), as you have done so with the ITC experiment.

RESPONSE: The co-IP experiments are quite massive to start (32 lanes in just the Figure 3 experiment!) with and therefore we couldn't include every single combination. However, we have included enough positive and negative treatments to allow for robust conclusions. In fact, all co-Ips in Figure 3 include a PiPexRD54 positive control.

Norwich Research Park
Norwich NR4 7UH
United Kingdom
+44 (0)1603 450 400
admin@TSL.ac.uk
www.TSL.ac.uk

In the discussion, the authors interestingly discuss the other biochemical roles that have been found for PexRD, and correctly suggest that by nature of it being evolutionary conserved, it is functional, and indeed evidence that it is expressed and localised in a similar manner across hosts supports that. But a main argument (or at least phrase) is it's an example of regressive evolution (in this case reduced ability to interact with ATG8). Given PexRD has a range of biochemical roles, could the loss of ATG8 binding co-inside with increased ability to perform one of those other roles the authors discuss, or perhaps even another unknown role? The word 'regressive' used throughout seems a little loaded given its defined as 'returning to a former or less developed state' – while presumably, this change is advantageous to P. mirablis. I imagine a gene swap experiment that shows P. mirablis is better able to infect its host using another species PexRD would support the notion its PexRD mutation(s) are actually regressive. I think some of these points would be good to clarify – and potentially rephrased.

RESPONSE: Indeed, regressive evolution here refers to the ATG8 binding activity and not any other potential activities of PmPexRD54, which is presumably functioning as a virulence effector. We have modified the text to address this point.

Reviewer #2: Same as above.

RESPONSE: Please see above.

Reviewer #3: 1. The authors conclude that the functionality of the PexRD54 AIM was lost in the P. mirabilis lineage, but does mutated PmPexRD54 contribute to virulence? For example, whether overexpress PmPexRD54 on M. jalapa have contribution to infection of P. mirabilis? Alternatively, does PmPexRD54 impair MjATG8s mediated autophagy function in Mirabilis plant? Do authors test anything on this?

RESPONSE: We would have loved to perform these hypotheses but the P. mirabilis/Mirabilis jalapa experimental is very limited and we don't have the transformation methods for both pathogen and host to do this.

2. Proposing the regressive evolution by using P. mirabilis PexRD54 AIM sequence example is fine. Whether mutated PexRD54 gain other function remain unclear and how many similar examples are not sure. There are several things are not sure. I suggest authors to shorten the speculation part in the manuscript.

RESPONSE: As discussed above, regressive evolution pertains to single traits as defined in the paper, and in this case is about the ATG8 binding activity and not any other functions of PmPexRD54. We have modified the text to clarify this point.

3. In S3 Fig, the transcription pattern of PmPexRD54 are different in three RNA-seq repeats, two of them showed that PmPexRD54 was not expressed in the early stage of infection(< 5dpi). One may ask the consistency of the experiment. It's better to show other Phytophthora genes as control and make a good explanation.

RESPONSE: Yes, we have noted that the expression pattern of PmPexRD54 is variable. We don't have an explanation for this, but we have noted variability in the P. mirabilis infection assays.

4. Please update the references 10, "A single amino acid polymorphism in a conserved effector of the multihost blast fungus pathogen expands host-target binding spectrum." has been published on PLOS Pathogens.

TSL

The Sainsbury Laboratory

Norwich Research Park
Norwich NR4 7UH
United Kingdom
+44 (0)1603 450 400
admin@TSL.ac.uk
www.TSL.ac.uk

RESPONSE: Modified as suggested.

The Sainsbury Laboratory is a registered charity number 1065510, and a registered company limited by guarantee number 3346853 in England & Wales
Registered office: John Innes Centre, Norwich Research Park, Norwich NR4 7UH, United Kingdom
Executive Director: Professor Nick Talbot FRS
